# 75 Tranexamic Acid Reduces Burn Wound Conversion and Lung Inflammation in a Murine Comb Burn Model

**DOI:** 10.1093/jbcr/irad045.049

**Published:** 2023-05-15

**Authors:** Damien Carter, Doreen Kacer, Elizabeth Turner, Igor Prudovsky

**Affiliations:** Maine Medical Center, Portland, Maine; Maine Health Institute for Research, Scarborough, Maine; Maine Medical Center, Portland, Maine; Maine Health Institute for Research, Scarborough, Maine; Maine Medical Center, Portland, Maine; Maine Health Institute for Research, Scarborough, Maine; Maine Medical Center, Portland, Maine; Maine Health Institute for Research, Scarborough, Maine; Maine Medical Center, Portland, Maine; Maine Health Institute for Research, Scarborough, Maine; Maine Medical Center, Portland, Maine; Maine Health Institute for Research, Scarborough, Maine; Maine Medical Center, Portland, Maine; Maine Health Institute for Research, Scarborough, Maine; Maine Medical Center, Portland, Maine; Maine Health Institute for Research, Scarborough, Maine

## Abstract

**Introduction:**

Burn wound conversion is a process where superficial partial thickness burns convert into deep partial and even full thickness burn injuries. Unfortunately, the pathophysiology of this phenomenon is poorly understood. A therapeutic intervention that may prevent secondary progression and cell death in burn-injured tissue is desirable. Recent work by our group has established that tranexamic acid (TXA) has significant anti-inflammatory properties in addition to its well-known anti-fibrinolytic effects. This study investigates tranexamic acid as a novel therapeutic treatment to mitigate burn wound conversion and reduce systemic inflammation.

**Methods:**

Sprague-Dawley rats were subjected to a hot comb burn contact injury using a 150-g brass comb preheated to 100°C, to create four rectangular burns, separated by three unburned interspaces. A subset of animals underwent the comb burn injury with an adjacent 30% TBSA contact injury using solid brass rods. The interspaces represent the ischemic zones simulating the zone of stasis. The treatment group received IP injection of TXA (100mg/kg) immediately after injury and once daily until sacrifice. Sham animals underwent an identical procedure, with application of a room temperature comb. Animals were sacrificed at 6hrs and 7-day time points post-injury. Photo images were obtained of the comb burn injury. Full-thickness biopsies from the ischemic zones and lung tissue were assessed with established histological and immunohistochemical (IHC) techniques.

**Results:**

At 7-days post injury, the percentage of ischemic zones with necrosis was significantly reduced in the treatment group when compared with untreated burn controls by photographic image analysis. When compared with controls, the treatment groups had significantly less progression of ischemic zones to necrosis when assessed by standard microscopy. These findings were consistent in both the comb burn only and comb burn + 30%TBSA injury models. The TXA treatment group demonstrated significantly more expression of the regeneration marker (CTHRC-1) and a trend toward increased expression of the Ki-67 proliferation marker when compared to untreated burn group. At 6 hours post-injury, TXA significantly decreased infiltration of neutrophils in the lungs for the 30%TBSA burn injury model. Furthermore, systemic levels of mitochondrial DNA, IL-1α and TNFα were significantly lower in TXA treated animals when compared to untreated burn controls.

**Conclusions:**

Animals treated with tranexamic acid demonstrated reduced burn wound conversion and decreased burn-induced SIRS response. Lung inflammation was also significantly reduced by administration of TXA after injury.

**Applicability of Research to Practice:**

Tranexamic acid and agents with similar mechanisms of action may provide a treatment to reduce burn wound conversion. This therapy could be easily incorporated into standard acute burn care practices.

